# ChromNetMotif: a Python tool to extract chromatin-sate marked motifs in a chromatin interaction network

**DOI:** 10.1093/bioadv/vbad126

**Published:** 2023-09-14

**Authors:** Benjamin Soibam

**Affiliations:** Department of Computer Science and Engineering Technology, University of Houston-Downtown, Houston, TX 77002, United States

## Abstract

**Motivation:**

Analysis of network motifs is crucial to studying the robustness, stability, and functions of complex networks. Genome organization can be viewed as a biological network that consists of interactions between different chromatin regions. These interacting regions are also marked by epigenetic or chromatin states which can contribute to the overall organization of the chromatin and proper genome function. Therefore, it is crucial to integrate the chromatin states of the nodes when performing motif analysis in chromatin interaction networks. Even though there has been increasing production of chromatin interaction and genome-wide epigenetic modification data, there is a lack of publicly available tools to extract chromatin state-marked motifs from genome organization data.

**Results:**

We develop a Python tool, ChromNetMotif, offering an easy-to-use command line interface to extract chromatin-state-marked motifs from a chromatin interaction network. The tool can extract occurrences, frequencies, and statistical enrichment of the chromatin state-marked motifs. Visualization files are also generated which allow the user to interpret the motifs easily. ChromNetMotif also allows the user to leverage the features of a multicore processor environment to reduce computation time for larger networks. The output files generated can be used to perform further downstream analysis. ChromNetMotif aims to serve as an important tool to comprehend the interplay between epigenetics and genome organization.

**Availability and implementation:**

ChromNetMotif is available at https://github.com/lncRNAAddict/ChromNetworkMotif.

## 1 Introduction

Studies show that in complex networks, a small set of network structures called network motifs exhibit much higher frequencies than expected (Lee *et al.* 2002, Milo *et al.* 2002, Shen-Orr *et al.* 2002). Network motifs are building blocks that can be used to understand and interpret a biological network (Milo *et al.* 2002, Sneppen *et al.* 2010). Different network motifs have been linked to various biological information processing (Luscombe *et al.* 2004) and unique responses to external signals to the networks. For example, one of the most widely studied motifs, the feedforward loop motif in directed networks, allows noise reduction function and network robustness to changes in environments (Ernst and Kellis 2010, Ocone *et al.* 2015). In undirected networks, motifs have been used to link function and topology in protein–protein interaction and transcription regulation networks (Vázquez *et al.* 2004, Song *et al.* 2017). Analysis of network motifs has also been used to study robustness and stability in undirected and directed complex networks (Angulo *et al.* 2015, Dey *et al.* 2019).

3D genome is organized into hierarchical modular units called topologically associated domains (TADs), which exhibit much higher levels of interactions inside the domain compared to interdomain interactions (Dixon *et al.* 2012, 2016). Genome organization allows the necessary interactions and loops between different regulatory regions to establish the proper gene and regulatory networks (Dixon *et al.* 2016, Zhang *et al.* 2019). Chromatin interactions regulate gene expression by bringing distal regulatory elements in close spatial proximity. Genome organization can be viewed as an undirected complex network G (E, V), where the set of nodes (V) consists of chromatin regions and E is the set of edges representing interactions between the different chromatin regions in V. In addition to interacting chromatin regions, epigenetic modifications of different regions in the chromatin mark them with chromatin states and are essential for the modulation of gene expression and many biological processes such as cell development, cell proliferation, and apoptosis (Ernst and Kellis 2010, Kundaje *et al.* 2015). The prediction of chromatin loops is dependent on the chromatin states of the participating chromatin regions(Wang *et al.* 2021). Since interacting regulatory elements in the chromatin which are a part of the genome organization should be marked with appropriate epigenetic modifications, it is essential to consider the chromatin state of the nodes in a network motif. When the chromatin states of the nodes are considered, a specific type of network motif can further be categorized into multiple chromatin state-marked motifs. Chromatin state-marked motifs can provide insights into the interplay between local epigenetic states and chromatin interactions.

## 2 Overview

ChromNetMotif is a tool that offers an easy-to-use Python command line interface to extract chromatin state-marked motifs from chromatin interaction network data (e.g. obtained from Hi-C) and chromatin states data (e.g. obtained from histone ChIP-Seq data). The tool can extract occurrences and frequencies of different chromatin state-marked motifs but also computes *P*-values to quantify the statistical enrichment of the motifs in the network. The statistical metrics are computed by comparing to several random networks the tools generate internally. Visualization files are also generated that allow the user to interpret the motifs. Because the statistical enrichment calculation can be computationally time intensive, ChromNetMotif contains a feature that allows simultaneous processing of random networks in a multicore processor environment. ChromNetMotif aims to serve as an important tool for researchers who aim to comprehend the interplay between epigenetics, genome organization, and gene regulation.

## 3 Features

### 3.1 Data import

**Figure 1.**
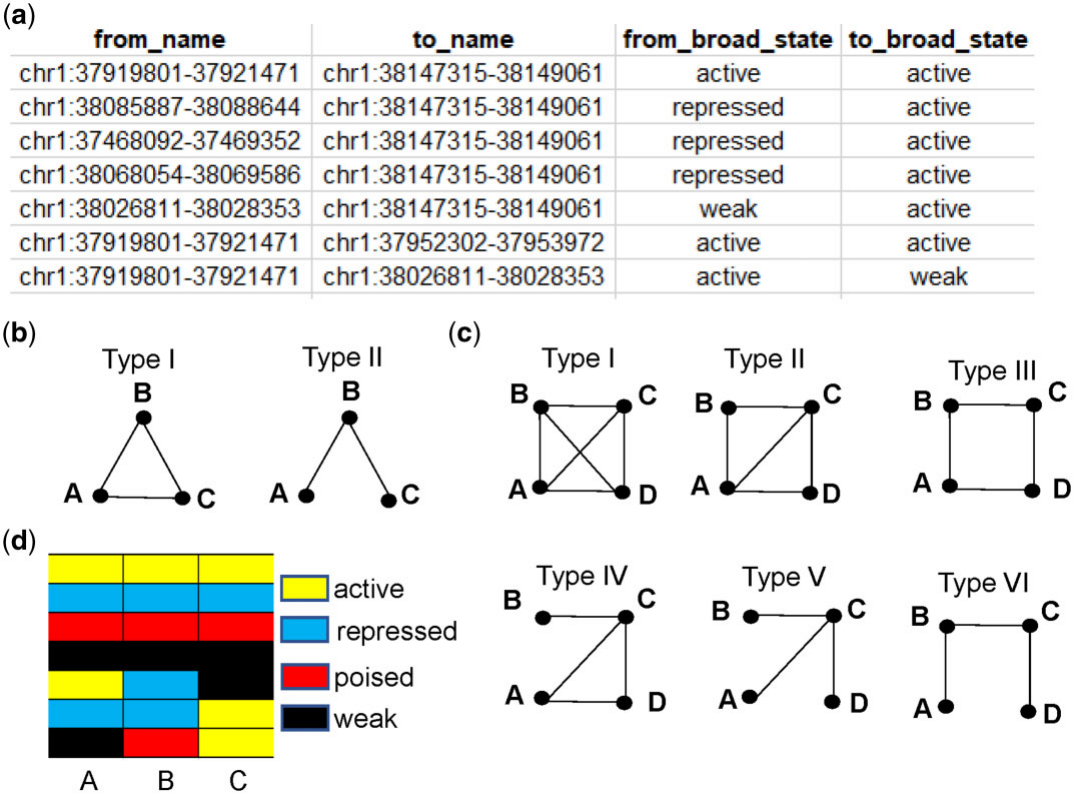
Chromatin state-marked network motifs. (a) An example chromatin state-marked chromatin interaction network file. Each row is an interaction with participating regions indicated in the first and second columns and their chromatin states in the third and fourth columns. (b) Possible network motifs of size 3 and (c) size 4 without considering the chromatin states of the nodes. Seven different possible chromatin state-marked Type I motif (size = 3) are shown in (d). Four possible chromatin states are considered. Participating nodes: A, B, and C can be marked as one of the four example chromatin states: “active,” “repressed,” “poised,” and “weak.”

ChromNetMotif requires an input chromatin-state network file in the form of a CSV file which contains four columns. The CSV should contain a header line. The header line contains the names of the four columns separated by commas. An example header line can be “from_node, to_node, from_broad_state, to_broad_state.” Each subsequent row in the file represents an edge in the chromatin network file with the first two columns representing the two interacting chromatin regions (or nodes in a network). The chromatin regions can be provided in “chromosome: start-end” format or a string that annotates the interacting region. An example file is shown in Fig. 1a. The third and fourth columns indicate the chromatin states of the interacting regions. The current version of ChromNetMotif can handle only undirected and unweighted networks. Note that the name of the header doesn’t matter as long as the column order is maintained.

### 3.2 Chromatin state-marked motifs finding

First, ChromNetMotif utilizes the subgraph finding tool gtrieScanner (Ribeiro and Silva 2010, 2012, 2014) to extract the occurrences of all possible network subgraphs of a specific size in the given network. For this, the chromatin states of the nodes in the subgraphs are not incorporated. Possible subgraphs of sizes 3 and 4 are shown in Fig. 1b and c. Next, the chromatin states of the nodes are incorporated in the extracted subgraphs to obtain subgraphs consisting of nodes marked with chromatin states. With the chromatin states assigned to the nodes, the original subgraphs are further grouped into different groups of subgraphs. Each group is a potential chromatin state-marked motif. For example, in the type I network motif of size 3 (Fig. 1b), the nodes are interconnected. If there are N unique chromatin states each node can take, there are three possible cases for the type I network motif of size 3: (a) all three nodes have the same chromatin state, (b) only two nodes have the same chromatin state, and (c) no two nodes have the same chromatin state. Cases (a), (b), and (c) will yield N, ^N^C_2_, and ^N^C_3_ possible chromatin state-marked motifs, respectively. If there are four possible chromatin states (e.g. active, repressed, poised, and weak), there can be 20 (4 + ^4^C_2_ + ^4^C_3_) different chromatin state-marked motifs of type I motif of size 3. Some example chromatin state-marked motifs are shown in Fig. 1d for four possible chromatin states (active, repressed, poised, and weak). The current version of ChromNetMotif can handle motifs of size 3 or 4. In future versions of ChromNetMotif, larger sizes of motifs will be incorporated.

ChromNetMotif computes the level of statistical enrichment of a chromatin state-marked motif by comparing the frequency of the chromatin state-marked motif of size k to that in randomized networks. An enriched motif in the real network should occur with lower frequency in a randomized network. We provide two different methods to generate random networks. In method I, a randomized network is obtained by performing a fixed number of double-edge swaps (swapping two edges in the network). A double-edge swap deletes two randomly chosen edges (e.g. edges a-b and x-y) in the network and creates the new edges a-x and b-y. The number of double-edge swaps is set to five times the number of edges in the network. The degree distribution in the randomized network is preserved compared to the real network. In method II, the network edges remain the same, but the chromatin states are randomized across the nodes in the network.

Using one of these two methods, several randomized networks (the default value is 500) are generated. In each randomized network, the frequency of the chromatin state-marked motif is counted. The number of randomized networks that had a greater or equal number of frequencies of the chromatin state-marked motif than the motif’s frequency in the real network is computed. The ratio of this number to the total number of randomized networks is the *P*-value of the chromatin state-marked network motif. A *P*-value threshold (for example .05) is used to filter statistically over-represented chromatin state-marked motifs in the real network. The over-represented motifs have higher occurrences in the real network compared to the averaged frequency in random networks. A minimum frequency count in the real network can also be used (in addition to the *P*-value) to filter the motifs. Besides, the fold change of the frequency in the real network compared to the mean frequency in random networks can be used to rank the motifs. The higher the fold change, the more enriched the motif is in the real network. To test if a particular chromatin state-marked motif is enriched in the real network, many random networks must be generated. To reduce the computational time, ChromNetMotif includes a feature that allows simultaneous analysis on different batches of the randomized networks across multiple cores. For a larger network size, one can leverage a multicore processor environment to reduce computation time.

### 3.3 Example analysis and output files

**Figure 2.**
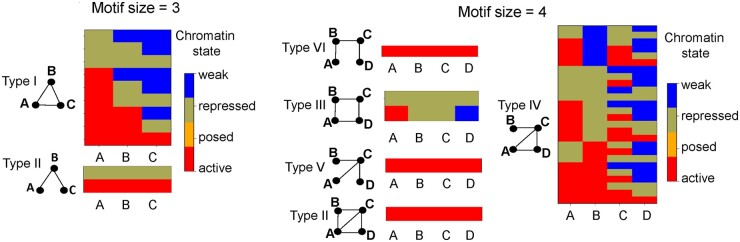
Chromatin state-marked motifs. The figure shows visualizations of examples of different chromatin state-marked motifs (of sizes 3 and 4) in the HeLa cell line. There are four possible chromatin states (“active,” “weak,” “poised,” and “repressed”) indicated by color codes. For example, in Type I of the motif of size 3, there are nine enriched different chromatin state-marked motifs as shown by the heatmap. In Type II of the motif of size 3, there are only two enriched different chromatin state-marked motifs as shown by the heatmap. The heatmaps for the motifs of size 4 are also shown.

ChromNetMotif generates a summary file that contains the chromatin state-marked motifs with the corresponding *P*-values, frequency in the real network, the mean frequency in randomized networks, and the logarithm of fold change of the frequency in the real network compared to the mean frequency in random networks. The chromatin states of the nodes in the motif are also provided. ChromNetMotif was applied to chromatin loop interaction data in the HeLa cell line (11 081 chromatin interactions and 12 021 nodes) obtained from a previous study (Wang *et al.* 2021). The chromatin state of the loop anchors was assigned one of the four states: active, repressed, weak, or poised based on the NIH epigenomics roadmap study (Kundaje *et al.* 2015) (Supplementary Methods). Using the randomization method I, ChromNetMotif identified 11 and 31 chromatin state-marked motifs of sizes 3 and 4, respectively (Supplementary Tables S1 and S2) with *P*-value < .05 and a minimum frequency requirement of 50 in the actual network. Motif types I and IV are the most prevalent motifs of sizes 3 and 4, respectively indicating a preferential occurrence of these specific repeating structures in the chromatin interaction network (Supplementary Tables S1 and S2 and Fig. 2). The top-ranked motif (based on the fold change) for these two most prevalent motif types have all the nodes marked as active (Supplementary Tables S1 and S2). In type I of motif size = 3, all the three chromatin regions interact with each other indicating that these three genomic regions are spatially close to each other, and they are all marked active. This suggests that for the formation of this specific type I motif structure, the chromatin state of the regions involved should be preferentially marked active. For motifs of size = 3, it should be noted type I is more complex but more prevalent compared to type II motif (which has a linear structure) indicating that only linear motifs should not be expected from chromatin interaction networks. We also analyzed motif detection by changing the randomization method II. The set of significant chromatin state-marked motifs identified using randomization method II was very similar (Supplementary Tables S3 and S4).

For each motif type, a visualization file in the form of a heatmap that represents the enriched chromatin state-marked motifs is generated. Example visualization from the chromatin network of motif size (3 and 4) are shown in Fig. 2. For example, the heatmap for motif type I and II of size 3 represents nine and two different chromatin state-marked motifs, respectively (Fig. 2). In type II motif of size 3, the chromatin states of all three nodes are either marked active or repressed (Fig. 2). In motif size 4, type IV motif has the most diverse set of chromatin state-marked motifs (Fig. 2). ChromNetMotif also generates an output file that contains all instances of the occurrences of the chromatin-state-marked motifs in the actual network. This file contains the names of the nodes (chromatin regions) from the original input chromatin state network file that participate in the enriched motifs. This will allow the user to perform downstream functional analysis of chromatin regions that participate in specific types of enriched chromatin state-marked motifs. For example, one can extract the closest genes to these enriched motifs and perform a gene ontology analysis on the genes. In this way, functional pathways associated with different types of chromatin state-marked motifs can be obtained. In the HeLa cell line, we extracted the chromatin locations of the motif type I of size 3 where all the three participating nodes are marked active. The closest genes to these locations were extracted and gene ontology was performed to obtain enriched GO terms (Supplementary Table S6). The top GO terms included cancer-related pathways such as “apoptotic process” and “inflammatory regulatory response.” This indicates a mediation of gene regulation via chromatin state-marked motifs in chromatin interaction networks.

In summary, ChromNetmotif addresses the need to extract chromatin state-marked motifs from complex genome organization networks, providing visualization of the motifs that can be interpreted easily. The output files generated can also be used to perform downstream analysis to uncover any links between the motifs and biological pathways.

## Supplementary Material

vbad126_Supplementary_Data
