# Supplementary material for: ChromNetMotif: a Python tool to extract chromatin-sate marked motifs in a chromatin interaction network
Source: Bioinform Adv. 2023 Sep 14;3(1):vbad126. doi: 10.1093/bioadv/vbad126 (PMC10517636; doi:10.1093/bioadv/vbad126)
Supplement: vbad126_Supplementary_Data [file vbad126_supplementary_data.docx]

**Supplementary Methods:**

Identifying Chromatin states of genome regions in CTCF loops:

The chromatin state data was obtained from NIH Roadmap Epigenomics Mapping Consortium (Kundaje *et al.*, 2015). The analysis divides the genome into a sequence of 200 bp long bins and assigns a “chromatin state” to each bin from one of the 18 possible states using a Hidden Markov Model. The 18 possible states were categorized into four broad categories: active, repressed, weak, and poised (Table S5). For each interacting region in the network, the number of bases overlapping with the four chromatin states was computed. The enrichment of each of the four-chromatin states was computed as (r/n)/(c/t), where r is the number of bases in the chromatin region overlapping with the chromatin state, n is the number of bases in the chromatin region, c is the total number of bases of the chromatin state, and t is the total number of bases of all the chromatin states. The chromatin state with the highest enrichment score was assigned as the chromatin state to the participating chromatin region. This normalization was done because the chromatin regions involved in the network do not represent the entire genome and hence assigning an epigenetic state to a node in the network should consider an abundance of the epigenetic state in the entire genome.

**Table S1: Chromatin-state marked motifs (of size =3) in the HeLa cell line using randomization method I.** The red colored row is not detected using randomization method II.

**Table S2: Chromatin-state marked motifs (of size =3) in the HeLa cell line using randomization method II.** The red colored row is not detected using randomization method I

**Table S3: Chromatin-state marked motifs (of size =4) in the HeLa cell line using randomization method I.**.

**Table S4: Chromatin-state marked motifs (of size =4) in the HeLa cell line using randomization method II.** The red colored row is not detected using randomization method I.

**Table S5: Categorization of 18 chromatin states into 4 broad chromatin states.**

| **STATE NO.** | **EPIGENETIC STATE** | **DESCRIPTION** | **BROAD STATE** |
| --- | --- | --- | --- |
| 1 | TssA | Active TSS | Active |
| 2 | TssFlnk | Flanking TSS | Active |
| 3 | TssFlnkU | Flanking TSS Upstream | Active |
| 4 | TssFlnkD | Flanking TSS Downstream | Active |
| 5 | Tx | Strong transcription | Active |
| 9 | EnhA1 | Active Enhancer 1 | Active |
| 10 | EnhA2 | Active Enhancer 2 | Active |
| 6 | TxWk | Weak transcription | Weak |
| 7 | EnhG1 | Genic enhancer1 | Weak |
| 8 | EnhG2 | Genic enhancer2 | Weak |
| 11 | EnhWk | Weak Enhancer | Weak |
| 14 | TssBiv | Bivalent/Poised TSS | Poised |
| 15 | EnhBiv | Bivalent Enhancer | Poised |
| 12 | ZNF/Rpts | ZNF genes & repeats | Repressed |
| 13 | Het | Heterochromatin | Repressed |
| 16 | ReprPC | Repressed PolyComb | Repressed |
| 17 | ReprPCWk | Weak Repressed PolyComb | Repressed |
| 18 | Quies | Quiescent/Low | Repressed |

**Table S6: Gene ontology analysis of genes closest to chromatin-state marked motif (size =3 and type I with all active marked nodes).**

**References**

1. Consortium, R. E. *et al.* Integrative analysis of 111 reference human epigenomes. *Nature* **518**, 317–330 (2015).

2. Kundaje, A. *et al.* Integrative analysis of 111 reference human epigenomes. *Nature* **518**, 317–330 (2015).
